# Beall's list of predatory open access journals: RIP

**DOI:** 10.1002/nop2.78

**Published:** 2017-02-28

**Authors:** Roger Watson

**Affiliations:** ^1^ Nursing Open

In January 2017, those visiting Jeffrey Beall's (http://scholarlyoa.com/) list of predatory online journals—called Scholarly Open Access—were disappointed to note: “This service is no longer available.” Searching the site on Google, while the original link to the page remained at the top, revealed a list of web pages and blogs reporting the closure of the service. Speculation about why the list was withdrawn continues. The official line from the University of Colorado—Beall's employer—was that the withdrawal was voluntary and that Beall would be focusing his scholarly activities elsewhere. However, other websites report that threats of legal action may have forced the decision.

For anyone who does not know, Beall's list was a website where information on the growth of online predatory journals, publishers and conference organisers was listed. It provided an easy checklist against the flood of uninvited emails from unknown publishers who send us “Greetings” and refer to us as “Esteemed” while outlining the purported benefits of sending them your manuscript. The benefits usually include quick and cheap open access publishing of your manuscript…if you are lucky. In some cases you will never see an article published but you will have parted with your money. I have addressed the problems associated with predatory publishers in a podcast (Watson, 2016a,b) and in past editorials in *Nursing Open* (Watson, 2015a,b,c, 2016a,b, 2017). Likewise, the *Journal of Advanced Nursing* has been very active in promoting good practice in open access publishing (Clark & Thompson, 2016; Darbyshire, McKenna, Lee, & East, 2016; Pickler et al., 2015).

At the fringes of the predatory publisher movement there is outright fraud and criminal activity. Otherwise, I imagine the bulk of these predators have seen a route to a “fast buck” by undercutting the established open access publishers who are very expensive. However, the problem with predatory publishers lies in their cheapness and speed which both obviate good peer review and editing as shown in at least one notable case (Mazières & Kohler, 2005). Beall's list was a “blacklist” of journals to be avoided. The nearest we have to an alternative is the DOAJ (Directory of Open Access Journals) which provides a “green tick” to those journals—such as *Nursing Open*—which have met some minimal criteria.

Admittedly, Beall's list was subjective and his methodology was not transparent. Nevertheless, in my experience of using it, he was rarely wrong. Occasionally I was surprised to see some journals included, and some journals were removed from the list on appeal. But these were never journals in the top echelons and they were, presumably, included in the list for good reason. If journals were removed from time to time and appeal was possible, it is hard to see how anyone could seriously challenge the existence and content of Beall's list. He may have made mistakes, but he challenged and changed bad behaviour and that has to have benefited authors and publishers. In my view, a valuable service has died and gone; will some Phoenix “arise from the ashes?”
